# Dysregulated bile acid metabolism as a novel player in gout progression: emerging therapeutic strategies

**DOI:** 10.3389/fendo.2025.1676017

**Published:** 2025-11-03

**Authors:** Hui Sun, Le Yang, Ye Sun, Xinya Zhang, Xin Sun, Xueping Zhao, Hui Sun, Qimeng Zhang, Guangli Yan, Xijun Wang

**Affiliations:** ^1^ State Key Laboratory of Dampness Syndrome of Chinese Medicine, The Second Affiliated Hospital of Guangzhou University of Chinese Medicine, Guangzhou, China; ^2^ State Key Laboratory of Integration and Innovation for Classic Formula and Modern Chinese Medicine, National Chinmedomics Research Center, Metabolomics Laboratory, Department of Pharmaceutical Analysis, Heilongjiang University of Chinese Medicine, Harbin, China

**Keywords:** gout, bile acid metabolism, uric acid homeostasis, NLRP3 inflammasome, gut-joint axis, FXR antagonists

## Abstract

Gout, a prevalent metabolic disorder driven by hyperuricemia, results in pathological deposition of monosodium urate (MSU) crystals in joints and soft tissues, stimulating intense inflammatory responses with systemic health consequences. Emerging evidence highlights dysregulated bile acid (BA) metabolism as a pivotal contributor to gout pathogenesis. Imbalances in BA influence disease progression through multiple mechanisms (1): modulating hepatic urate production via PPAR-α/XOD signaling (2), regulating immune responses through FXR/TGR5-dependent suppression of NLRP3 inflammasome activation, and (3) shaping the gut microbiota composition, which reciprocally affects uric acid homeostasis and inflammation. Despite these advances, the precise mechanistic networks linking BA dysmetabolism to gout remain incompletely understood. In this review, we systematically synthesizes current knowledge on BA-gout interactions, elucidated how BA disturbances exacerbate disease progression, discussed the factors contributing to metabolic disorders of BAs, and evaluated promising therapeutic strategies targeting BA pathways. For example, FXR antagonists facilitate the synthesis of BA by inhibiting the aberrant activation of FXR. TGR5 agonists suppress inflammation. Probiotics help restore the diversity of the gut microbiota and increase the abundance of beneficial bacteria, including *Bifidobacterium* and *Lactobacillus*. Moreover, traditional Chinese medicine works by improving structural disorders of the gut microbiota and activating CYP7A1 to enhance the BA synthesis pathway. By integrating metabolic, immunological, and microbial perspectives, this work provides a framework for developing novel, mechanism-based interventions against gout.

## 1 Introduction

Gout, a prevalent metabolic disorder characterized by hyperuricemic-induced monosodium urate (MSU) crystal deposition in joints and periarticular tissues, elicits intense inflammatory responses that cause significant morbidity (1, 2). In China, gout affects ~1.1% of the population (>16 million cases), with prevalence rising parallel to the surge in hyperuricemia (14.0% in 2019 vs. 11.1% in 2015) (3, 4). As the second most common metabolic disease after diabetes (5), gout manifests as acute arthritis (erythema, swelling, and debilitating pain) and, if untreated, progresses to chronic tophaceous joint destruction, nephrolithiasis, and cardiovascular complications (6–11). The available therapeutic agents (colchicine, non-steroidal anti-inflammatory (NSAIDs) and urate-lowering drugs) primarily alleviate symptoms but fail to modify disease progression and often cause adverse effects (12), underscoring the need for novel therapeutic strategies targeting underlying pathogenesis. Emerging evidence implicates bile acid (BA) metabolism as a critical regulator of gout pathophysiology. BAs, classically known for lipid digestion, are now recognized as pleiotropic signaling molecules modulating metabolic homeostasis, inflammation, and gut microbiota (13–15). Dysregulated BA profiles are linked to metabolic disorders including non-alcoholic fatty liver disease (NAFLD), diabetes, and atherosclerosis (16, 17), with reciprocal crosstalk between inflammation and BA synthesis (e.g., IL-1β-mediated suppression of cholesterol 7α-hydroxylase (CYP7A1)) (18, 19). Intriguingly, gout patients exhibit reduced BA synthesis and altered BA pools (20, 21), suggesting that BA dysmetabolism may orchestrate gout progression through multiple mechanisms: 1) Uric acid (UA) metabolism: BAs inhibit hepatic xanthine oxidase (XOD) via proliferator-activated receptor alpha (PPAR-α), reducing urate production; their deficiency exacerbates hyperuricemia (22). Impaired FXR activation due to low BAs also disrupts lipid metabolism, further diminishing renal urate excretion. Moreover, intestinal FXR deficiency increases intestinal XOD activity, resulting in higher UA production. 2) Inflammation: BA-activated FXR/TGR5 pathways suppress the NLRP3 inflammasome and release of cytokine (IL-1β and TNF-α) (23–25). The depletion of BAs in gout patients exacerbates MSU crystal-driven inflammation via unchecked immune cell (T lymphocyte/neutrophil/macrophage) activation. 3) Gut microbiota: Antimicrobial BAs shape microbial composition; their reduction in gout patients enriches pathobionts (e.g., *Escherichia coli* and *Bacteroides*) that promote urate accumulation and inflammation (26–28).

In this review, we synthesized existing information on BA-gout interactions, highlighting: the mechanistic interplay between BA dysregulation and gout pathogenesis across metabolic, immune, and microbial axes; the therapeutic potential of BA-targeted interventions (FXR antagonists, TGR5 agonists, and microbiota modulation) to concurrently address hyperuricemia and inflammation; and the clinical implications for gout management strategies. By elucidating BA-centric pathways, we aimed to advance gout therapeutics beyond symptomatic relief toward disease modification.

## 2 BA homeostasis in gout pathogenesis

### 2.1 Clinical manifestations

Studies have indicates that patients with hyperuricemia commonly exhibit impaired BA homeostasis (29). In male patients, serum unconjugated BA levels were significantly lower in the hyperuricemia group compared to the non-hyperuricemia group. Specifically, chenodeoxycholic acid (CDCA) levels in the hyperuricemia group were 34.1% lower than those in the non-hyperuricemia group (21). Gout patients have lower BA levels. A clinical study analyzing serum metabolites from 31 gout patients and 31 healthy controls revealed significantly decreased levels of 3α,7α-dihydroxycoprostanic acid, 7α-hydroxycholesterol, and 27-deoxy-5β-cyprinol in gout patients, indicating impaired primary BA biosynthesis (20).

### 2.2 Role of dysregulated BA metabolism in lipid metabolism

Reduced BA levels contribute to excessive lipid accumulation. Alterations in BA composition and concentration may impair the activity of hepatic FXR and TGR5 (30). CDCA is the most potent FXR agonist among BAs, followed by deoxycholic acid (DCA) = lithocholic acid (LCA) > cholic acid (CA) (31). Gout patients exhibit decreased CDCA levels, leading to weakened FXR activity. Furthermore, decreased BA levels attenuate the activation of FXR. Activation of FXR inhibits the expression of sterol regulatory element-binding protein 1c (SREBP-1c) by enhancing small heterodimer partner (SHP) expression (32), and reduces the activity of lipogenic enzymes such as fatty acid synthase (FAS), acetyl-CoA carboxylase (ACC), and stearoyl-CoA desaturase-1 (SCD-1), thereby lowering triglyceride levels (32, 33). Additionally, FXR activation stimulates the expression of PPAR-α, a key regulator of triglyceride metabolism, thereby inhibiting hepatic lipid accumulation and enhancing fatty acid β-oxidation efficiency (34, 35). Consequently, reduced BA levels in gout impair the function of FXR receptors. A decrease in FXR activity reduces fatty acid β-oxidation capacity, leading to excessive lipid accumulation. These changes increase the risk of obesity and insulin resistance, resulting in dysregulated lipid metabolism and an increase in inflammation.

**Figure 1:**
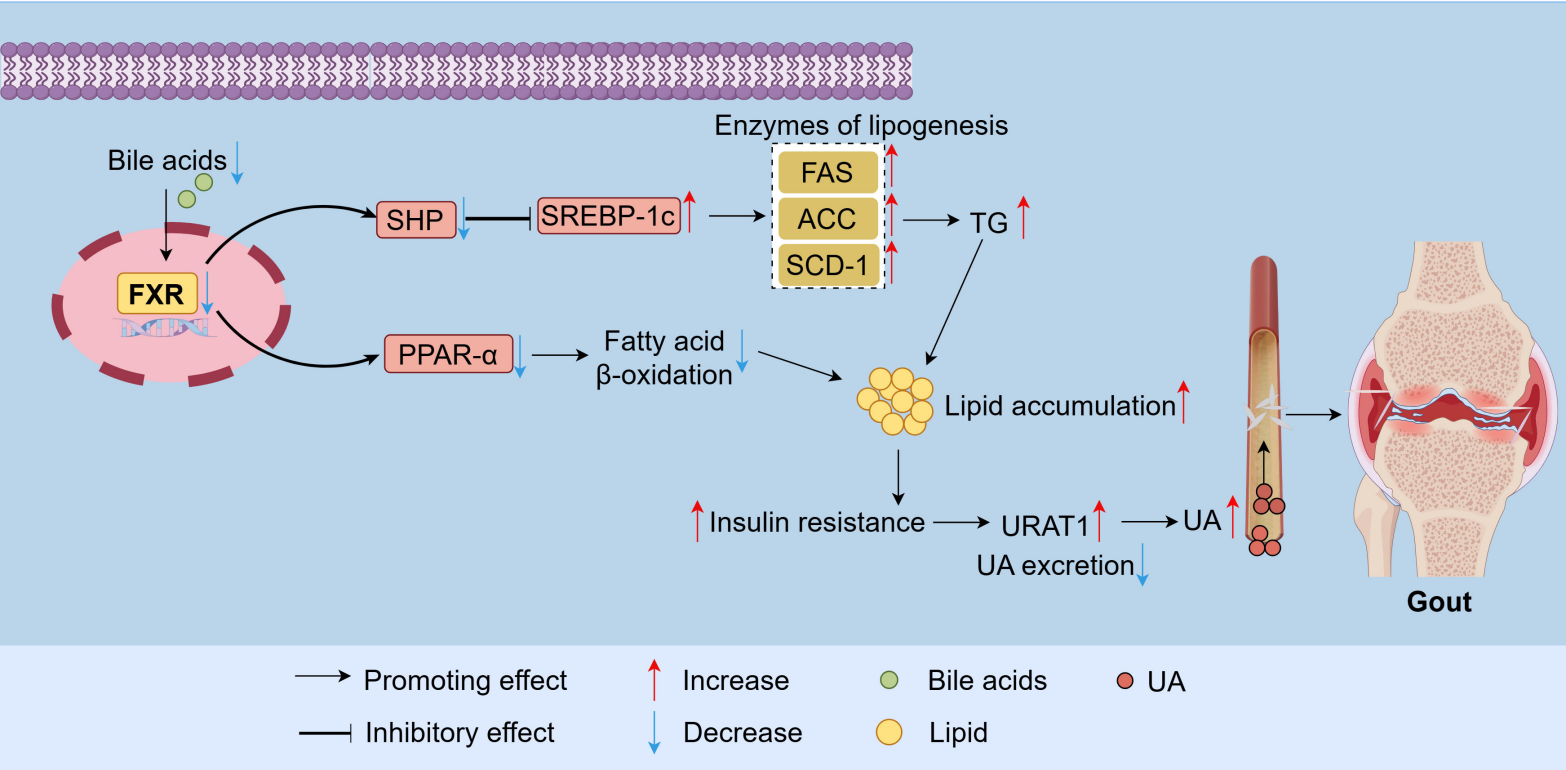
Dysregulated BA metabolism in gout disrupts lipid metabolism and impairs uric acid excretion. A decrease in BA levels attenuates FXR activity. FXR promotes hepatic TG synthesis and reduces fatty acid β-oxidation by upregulating the expression of SREBP-1c and inhibiting PPAR-α. This leads to the accumulation of lipids and metabolic disorders, thereby increasing serum UA levels. FXR, Farnesoid-X-receptor; SHP, Small heterodimer partner; PPAR-α, Proliferator-activated receptor alpha; SREBP-1c, Sterol regulatory element-binding protein 1c; TG, Triglyceride; FAS, Fatty acid synthase; ACC, Acetyl-CoA carboxylase; SCD-1, Stearoyl-CoA desaturase-1; UA, Uric acid; URAT1, Urate transporter 1.

Excessive lipid accumulation contributes to elevated UA levels in gout patients. This lipid overload can induce insulin resistance, which further enhances renal urate reabsorption by activating the urate transporter 1 (URAT1) and sodium-dependent anion cotransporters in the proximal renal tubules. Consequently, renal UA clearance decreases (36), thereby increasing serum UA levels (37) and accelerating gout progression (**Figure 1**).

### 2.3 Role of dysregulated BA metabolism in immune regulation

#### 2.3.1 T lymphocytes

**Figure 2:**
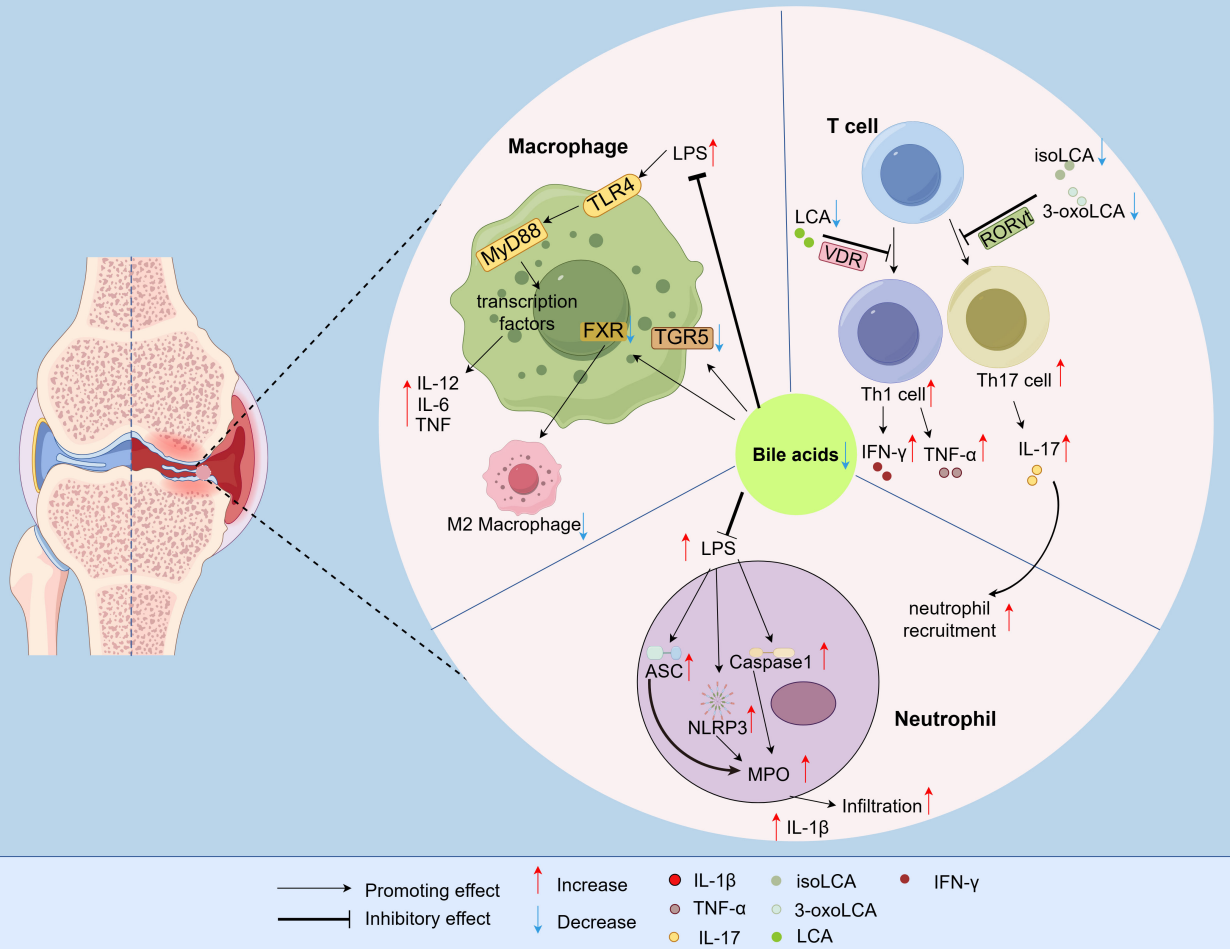
Immune cell regulation by BA metabolic dysregulation in gout. BAs constrain T-cell differentiation, block neutrophil infiltration, and dampen macrophage-induced inflammation, thereby attenuating inflammatory responses. In gout, a decrease in BA levels compromises this suppressive function, resulting in pathological inflammation that propagates disease progression. 3-oxoLCA, 3-oxolithocholic acid; isoLCA, iso-lithocholic acid; RORγt, Retinoic acid receptor-related orphan receptor gamma t; VDR, Vitamin D receptor; FXR, Farnesoid-X-receptor; TGR5, Takeda G protein-coupled receptor 5; TLR4, Toll-like receptor 4; MyD88, Myeloid differentiation primary response gene 88; LPS, Lipopolysaccharide; MPO, Myeloperoxidase; NLRP3, NOD-, LRR- and pyrin domain-containing protein 3; ASC, Apoptosis-associated speck-like protein containing a CARD; Caspase-1, Cysteine-aspartic ase-1; IFN-γ, Interferon-γ; IL-1β, Interleukin-1β; TNF-α, Tumor Necrosis Factor-α; IL-6, Interleukin-6; IL-12, Interleukin-12; IL-17, Interleukin-17.

BAs attenuate gout inflammation by inhibiting T cell differentiation and activation. Specifically, the BA metabolites 3-oxolithocholic acid (3-oxoLCA) and iso-lithocholic acid (isoLCA) impede Th17 cell differentiation by directly binding to retinoic acid receptor-related orphan receptor gamma t (RORγt), a key transcriptional promoter of Th17 cells (38, 39). IL-17, a pro-inflammatory cytokine secreted by Th17 cells, plays a key role in gout pathogenesis (40, 41). Additionally, LCA interacts with the vitamin D receptor (VDR) to inhibit the activation of Th1 cells, thereby reducing the production of Th1 cytokines such as IFN-γ and TNF-α (42). Consequently, reduced BA levels promote the production of pro-inflammatory cytokines (IL-17 and TNF-α), thereby exacerbating inflammatory responses in gout (**Figure 2**).

#### 2.3.2 Neutrophils

BAs suppress neutrophil infiltration into inflammatory sites, thereby inhibiting the onset of acute gout. Myeloperoxidase (MPO) activity serves as a biomarker for neutrophil infiltration at inflammatory loci. Studies demonstrate that BAs attenuate lipopolysaccharide (LPS)-induced upregulation of NLRP3, ASC, and Caspase-1, thereby reducing MPO activity, inhibit neutrophil infiltration, and alleviate inflammatory responses (43). In contrast, diminished BA levels promote Th17 differentiation, leading to greater production of the pro-inflammatory cytokine IL-17, which enhances neutrophil recruitment (44). Neutrophils are known to produce IL-1β, a key pro-inflammatory cytokine responsible for initiating acute gout attacks characterized by joint redness, swelling, warmth, and pain (45) (**Figure 2**).

#### 2.3.3 Macrophages

BAs attenuate inflammatory responses in gout by suppressing macrophage-mediated inflammation. Macrophages play an important role in the initiation, progression, and resolution of gout (46). It is reported that LPS activates the TLR4 receptor, which signals through MyD88, followed by the activation of different transcription factors, resulting in expression of pro-inflammatory (47). Studies have shown that BAs reduce the LPS-induced expression of pro-inflammatory cytokines, such as IL-6, tumour necrosis factor (TNF) and IL-12 in human macrophages (48). Additionally, the activation of TGR5 and FXR (receptors for BAs) inhibits the effector functions of macrophages and promotes their polarization toward the anti-inflammatory M2 phenotype (49). Consequently, a decrease in BA levels lead to enhanced pro-inflammatory cytokine production and impaired polarization towards the M2 anti-inflammatory phenotype, thereby exacerbating inflammatory responses in gout (**Figure 2**).

This chapter delineates the function of BA homeostasis in the pathogenesis of gout. We investigated how the dysregulation of BA metabolism can precipitate lipid metabolic disorders in gout patients, contribute to increased UA levels, and stimulate the production of pro-inflammatory factors by immune cells, including T cells, macrophages, and neutrophils, thereby exacerbating the progression of the disease. We subsequently examined the mechanisms underlying BA metabolic dysregulation in gout, building upon existing research.

## 3 Mechanisms underlying the dysregulation BA metabolism in gout

### 3.1 BA synthesis

BA synthesis in the liver occurs via two pathways: the classical pathway and the alternative pathway (50). The classical pathway is initiated by CYP7A1, the rate-limiting enzyme in primary BA synthesis. This cytochrome P450 enzyme catalyzes the conversion of cholesterol to 7α-hydroxycholesterol (51), and subsequently converted to CA through the action of cytochrome P450 12α-hydroxylase B1 (CYP8B1) and mitochondrial cytochrome P450 27A1 (CYP27A1). The intermediates are instead converted into CDCA when CYP8B1 is lacking (52). CYP8B1 is the essential enzyme for CA synthesis (53, 54). Next, 7α-hydroxycholesterol undergoes a series of modifications to generate CA and CDCA (55). This classical pathway primarily produces CA and CDCA (56, 57). The alternative pathway is initiated by CYP27A1. The intermediate 27-hydroxycholesterol undergoes 7α-hydroxylation by oxysterol 7α-hydroxylase (cytochrome P450 family 7 subfamily B member 1 (CYP7B1)), followed by a series of modifications to produce CDCA (58, 59).

**Figure 3:**
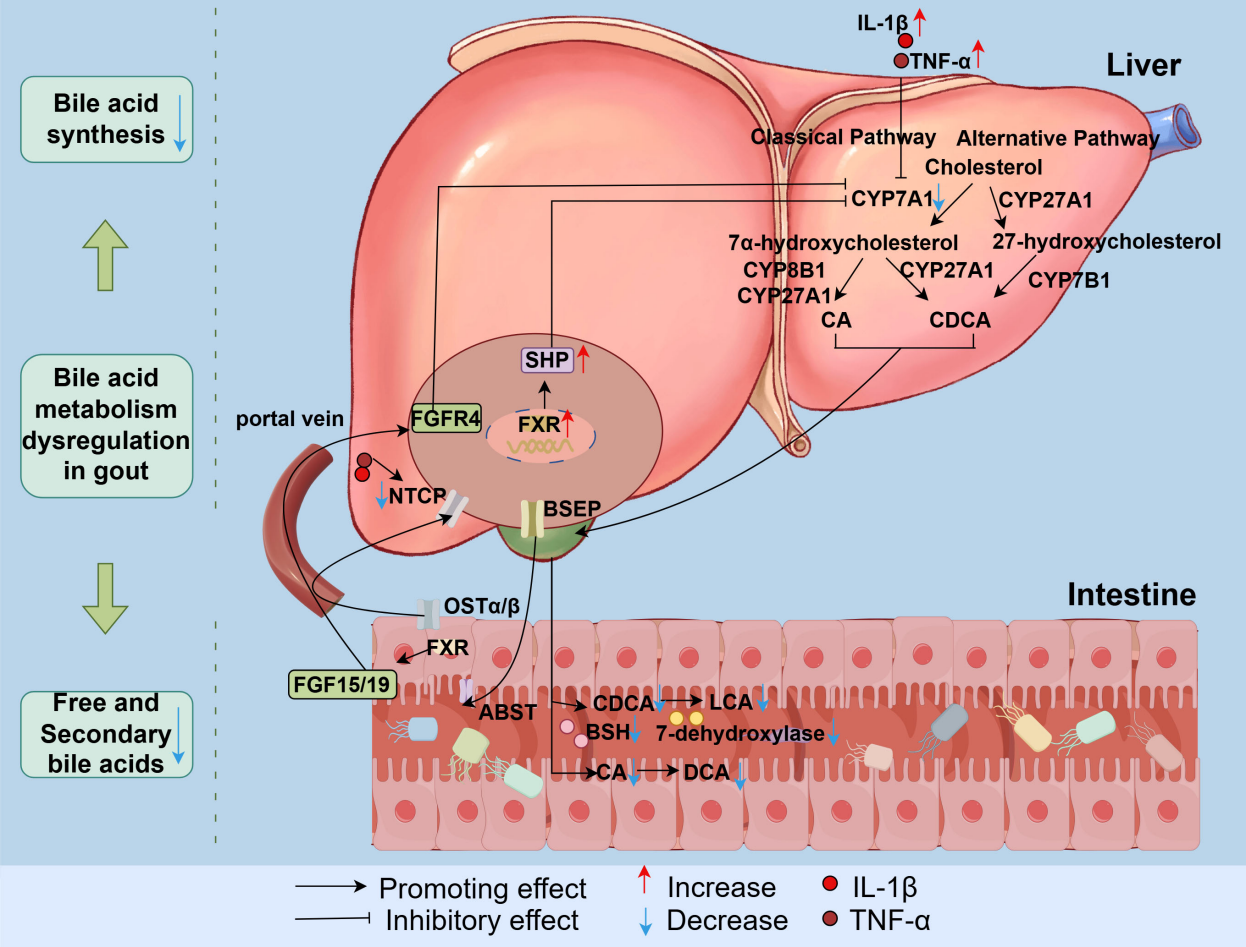
Mechanisms underlying BA metabolism dysregulation in gout. In gout, dysregulated hepatic FXR activation combined with elevated IL-1β and TNF-α levels collectively suppresses CYP7A1 expression, thereby reducing BA synthesis. Concurrently, an increase in the production of hepatic pro-inflammatory cytokines (TNF-α and IL-1β) downregulates the transcription of NTCP, impairing the function of NTCP and decelerating BA enterohepatic circulation. Furthermore, gout-associated gut dysbiosis decreases microbial consortia producing BSH and 7α-dehydroxylase, leading to reduced free and secondary BA proportions. BSH, bile salt hydrolase. CA, Cholic acid; CDCA, Chenodeoxycholic acid; LCA, Lithocholic acid; DCA, Deoxycholic acid; SHP, Small heterodimer partner; FXR, Farnesoid-X-receptor; IL-1β, Interleukin-1β; TNF-α, Tumor Necrosis Factor-α; CYP8B1, Cytochrome P450 12α-hydroxylase B1; CYP7A1, Cholesterol 7 alpha-hydroxylase; CYP27A1, Cytochrome P450 27A1; CYP7B1, Cytochrome P450 family 7 subfamily B member 1; BSEP, Bile salt export pump; ASBT, Apical sodium-dependent bile acid transporter; OSTα/β, Organic solute transporter alpha/beta; NTCP, Sodium taurocholate cotransporting polypeptide; FGF15/19, Fibroblast growth factor 15/19; FGFR4, Fibroblast growth factor receptor 4; BSH, Bile salt hydrolase.

In the gout state, abnormal activation of FXR and pro-inflammatory cytokines can suppress BA production, leading to the dysregulation of BA metabolism. Aberrant activation of hepatic FXR in gout inhibits the expression of CYP7A1. This reduces the conversion rate of cholesterol to BAs, consequently increasing cholesterol levels and decreasing the synthesis of BA (29). A clinical study revealed impaired primary BA biosynthesis and confirmed reduced BA biosynthesis in the gout state (20). Furthermore, IL-1β suppresses CYP7A1 transcription in human hepatocytes via the c-Jun N-terminal kinase/c-Jun signaling pathway (19). TNF-α significantly inhibits the expression of the CYP7A1 protein in hepatocytes (F = 47.92, P < 0.01) (60), thereby reducing BA synthesis (**Figure 3**).

### 3.2 BA transportation

Multiple transport proteins facilitate the circulation of BAs between the liver and intestines. For example, most synthesized free BAs are conjugated to glycine or taurine and are subsequently secreted into the gallbladder via the bile salt export pump (BSEP) and multidrug resistance-associated protein 2 (MRP2) (61). In the intestine, conjugated BAs are metabolized by the gut microbiota to generate secondary BAs (50, 62). About 95% of BAs in the intestinal lumen are subsequently reabsorbed primarily in the terminal ileum by the apical sodium-dependent bile acid transporter (ASBT). The reabsorbed BAs are then transported across enterocytes to the portal circulation by the organic solute transporter alpha/beta (OSTα/β) located on the basolateral membrane. These BAs return to the liver via the portal vein and re-enter hepatocytes under the action of the sodium taurocholate cotransporting polypeptide (NTCP) (13, 63, 64) (**Figure 3**). About 5% of BAs are excreted in feces. Under physiological conditions, the size of the BA pool remains stable through the coordinated function of this enterohepatic transport system (65).

In the gout state, UA induces endoplasmic reticulum (ER) stress in hepatic cells, activating SREBP-1c. This triggers overexpression of lipogenic enzymes, increasing intrahepatic fat production and promoting hepatic inflammation (66, 67). An increase in the production of hepatic pro-inflammatory cytokines (TNF-α and IL-1β) reduces the transcription of the NTCP gene, consequently decreasing both NTCP mRNA and protein levels. This ultimately impairs the function of NTCP and reduces the enterohepatic circulation rate of BAs (68, 69).

### 3.3 BA targets

#### 3.3.1 FXR

In the gout state, aberrant activation of hepatic FXR decreases BA synthesis. FXR is a nuclear receptor activated by BAs. Its primary function involves regulating BA synthesis and enterohepatic circulation to maintain BA homeostasis (70). In the liver, the activation of FXR suppresses BA synthesis while promoting BA transport into bile ducts (71). FXR directly activates transcription of the SHP, which subsequently negatively regulates the expression of CYP7A1. In the intestine, FXR plays a crucial role in enterohepatic circulation. Upon activation in the ileum, FXR induces the release of fibroblast growth factor 15 (FGF15). This enterokine reaches the liver via the enterohepatic circulation, where it binds to the fibroblast growth factor receptor 4 (FGFR4). The FGF19-FGFR4 complex then inhibits the expression of hepatic CYP7A1 (72, 73), ultimately suppressing BA synthesis (**Figure 3**).

**Figure 4:**
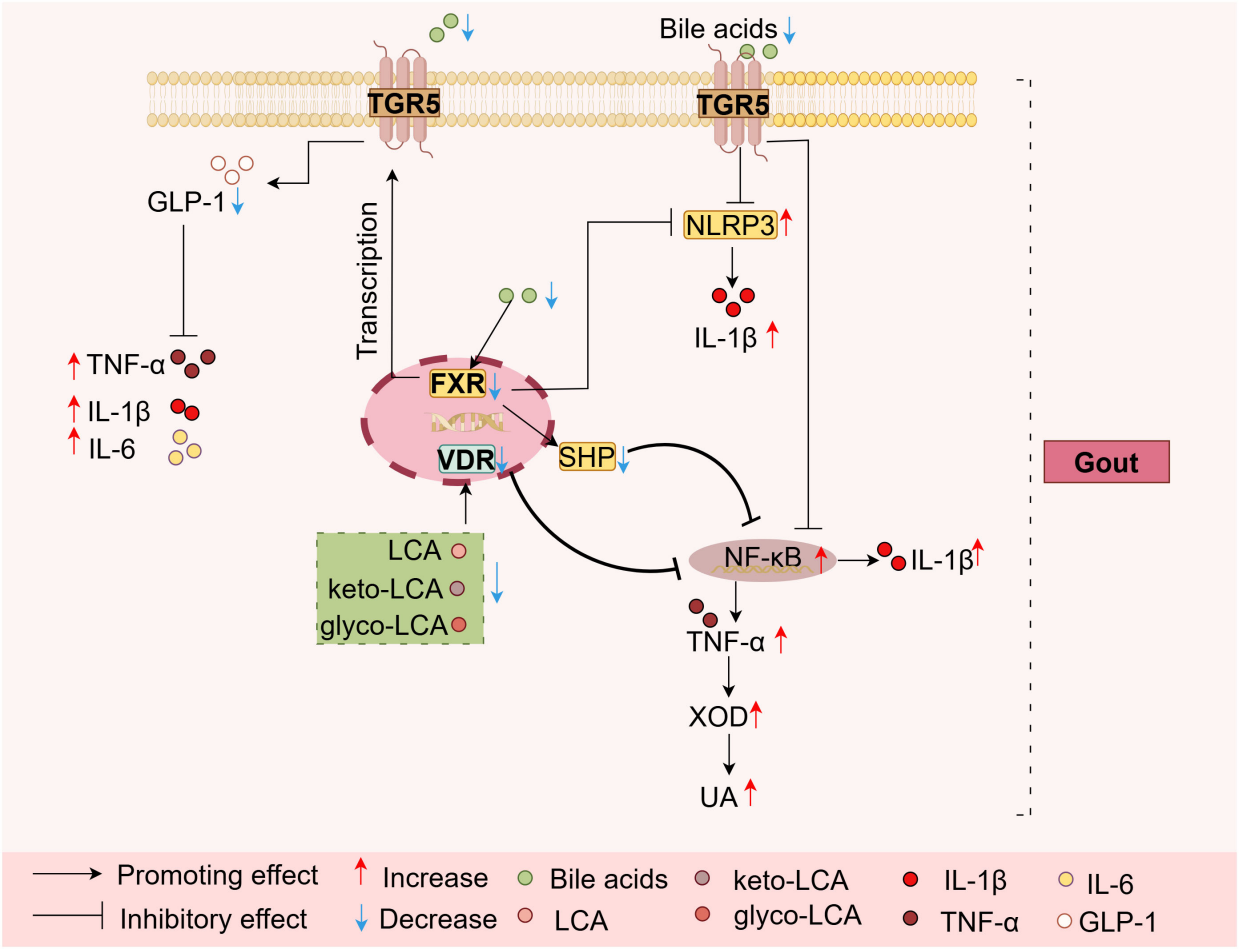
The roles of BA target FXR, TGR5, and VDR in gout BA metabolism disorders. A reduction in BA levels weakens the signaling of receptors FXR, TGR5, and VDR. This attenuation subsequently reduces the inhibitory tone on key inflammatory pathways, NLRP3 and NF-κB, thereby further promoting the generation of pro-inflammatory cytokines such as IL-1β and TNF-α. Furthermore, crosstalk exists between FXR and TGR5. FXR activation induces TGR5 gene transcription, and the activation of either receptor promotes the secretion of GLP-1 from intestinal L cells. The released GLP-1, in turn, exerts anti-inflammatory effects by attenuating the production of inflammatory cytokines, including TNF-α, IL-6, and IL-1β, forming a potential negative feedback loop. Notably, the elevated local TNF-α level, which is partly driven by intestinal FXR deficiency, induces the overexpression and hyperactivity of intestinal XOD, leading to increased intestinal uric acid synthesis. Consequently, the dysregulation of bile acid metabolism modulates FXR, TGR5, and VDR receptors, thereby enhancing inflammatory responses and uric acid synthesis, which exacerbates the progression of gout. FXR, Farnesoid-X-receptor; SHP, Small heterodimer partner; IL-1β, Interleukin-1β; TNF-α, Tumor Necrosis Factor-α; IL-6, Interleukin-6; UA, Uric acid; VDR, Vitamin D receptor; TGR5, Takeda G protein-coupled receptor 5; XOD, Xanthine oxidase; LCA, Lithocholic acid; glyco-LCA, Glycolithocholic acid; keto-LCA, Ketolithocholic acid.

Activation of FXR can inhibit the assembly of the NLRP3 inflammasome and, through the activation of SHP, suppress the NF-κB pathway. This process consequently reduces the production of pro-inflammatory factors (e.g., IL-1β, TNF-α) and mitigates inflammation (23, 49, 74, 75). Furthermore, research has demonstrated that increased local TNF-α level driven by intestinal FXR deficiency induces the overexpression and hyperactivity of intestinal XO, leading to elevated intestinal uric acid synthesis, ultimately resulting in hyperuricemia (21) (**Figure 4**).

In the context of gout, a decrease in BA levels leads to attenuated FXR activity, consequently diminishes the inhibitory effect on NLRP3 and NF-κB signaling pathways and promotes enhanced activity and expression of intestinal XOD, thereby triggering inflammatory responses and elevated UA levels, which exacerbate the progression of gout (**Figure 4**).

#### 3.3.2 TGR5

TGR5 is a G protein-coupled receptor activated by BAs. LCA is the most potent TGR5 agonist, followed by DCA, CDCA, CA (76). Notably, there exists crosstalk between FXR and TGR5, and FXR activation induce TGR5 gene transcription, thereby promoting glucagon-like peptide-1 (GLP-1) secretion from intestinal L cells (77, 78). Moreover, TGR5 activation also induces the release of GLP-1. Studies indicate that GLP-1 attenuates the production of the inflammatory cytokines TNF-α, IL-6, and IL-1β, thereby exerting anti-inflammatory effects (13, 79, 80). Furthermore, TGR5 activation inhibits the phosphorylation of IκBα in macrophages, thereby suppressing the NF-κB pathway. It also increases ubiquitination of the NLRP3 inflammasome, collectively dampening inflammatory responses (81, 82).

Conversely, in gout, reduced bile acid levels lead to diminished TGR5 activity, which in turn results in reduced GLP-1 secretion, and weakened inhibition of both the NF-κB pathway and NLRP3 inflammasome. These changes collectively promote inflammatory responses, thereby exacerbating the progression of gout (**Figure 4**).

#### 3.3.3 VDR

The nuclear receptor VDR is activated by BAs. VDR can be activated by BAs such as LCA, glycolithocholic acid (glyco-LCA), and ketolithocholic acid (keto-LCA) (83). VDR participates in the regulation of inflammatory responses. BAs exert anti-inflammatory effects by activating the VDR receptor to inhibit NF-κB signaling and suppress TNF-α expression (84).

In gout, diminished levels of secondary BAs lead to decreased VDR activation, which results in attenuated inhibition of NF-κB signaling. This effect ultimately disrupts bile acid synthesis and promotes inflammatory responses, thereby accelerating the progression of gout (**Figure 4**).

### 3.4 Gut microbiota dysbiosis

Gut microbiota dysbiosis in gout lead to reduced production of both free and secondary BAs. Gout patients have a greater abundance of *Bacteroides (* 85) along with decreased levels of *Bifidobacterium* and *Lactobacillus (* 26, 27). Additionally, hyperuricemic mice present reduced abundances of beneficial *Clostridium* bacteria and *Lactobacillus (* 86, 87), and hyperuricemic rats present decreased abundances of *Lactobacillus*, *Streptococcus*, and *Clostridium (* 88). The intestinal microbiota including *Lactobacillus*, *Lactococcus*, *Streptococcus*, *Bacillus*, *Enterococcus*, *Bifidobacterium*, and *Bacteroides*, expresses bile salt hydrolase (BSH) (89–91). This enzyme converts conjugated BAs to free forms by hydrolyzing the C24 N-acyl amide bond, which links BAs to their amino acid conjugates (taurine or glycine) (92). The secondary BAs generated through BSH-mediated deconjugation exhibit potent bactericidal activity (93, 94), thereby diversifying the structure and function of BAs while facilitating their fecal excretion (95). *Clostridium* species play a crucial roles in dehydroxylation, producing bile acid 7α-dehydroxylase, which converts primary BAs (CA and CDCA) into secondary BAs (DCA and LCA) (96, 97). Reduced abundance of *Clostridium* results in diminished synthesis of secondary BAs (98). *Clostridium* strain S2 produces sulfatase enzymes that catalyze the desulfation of BAs. Bacterial desulfation facilitates the reabsorption of BAs and is essential for maintaining homeostasis of the BA pool (30). As evidenced above, gut dysbiosis in gout reduces microbial populations producing BSH and 7α-dehydroxylase, leading to a decreased proportion of free and secondary BAs (**Figure 3**).

**Figure 5:**
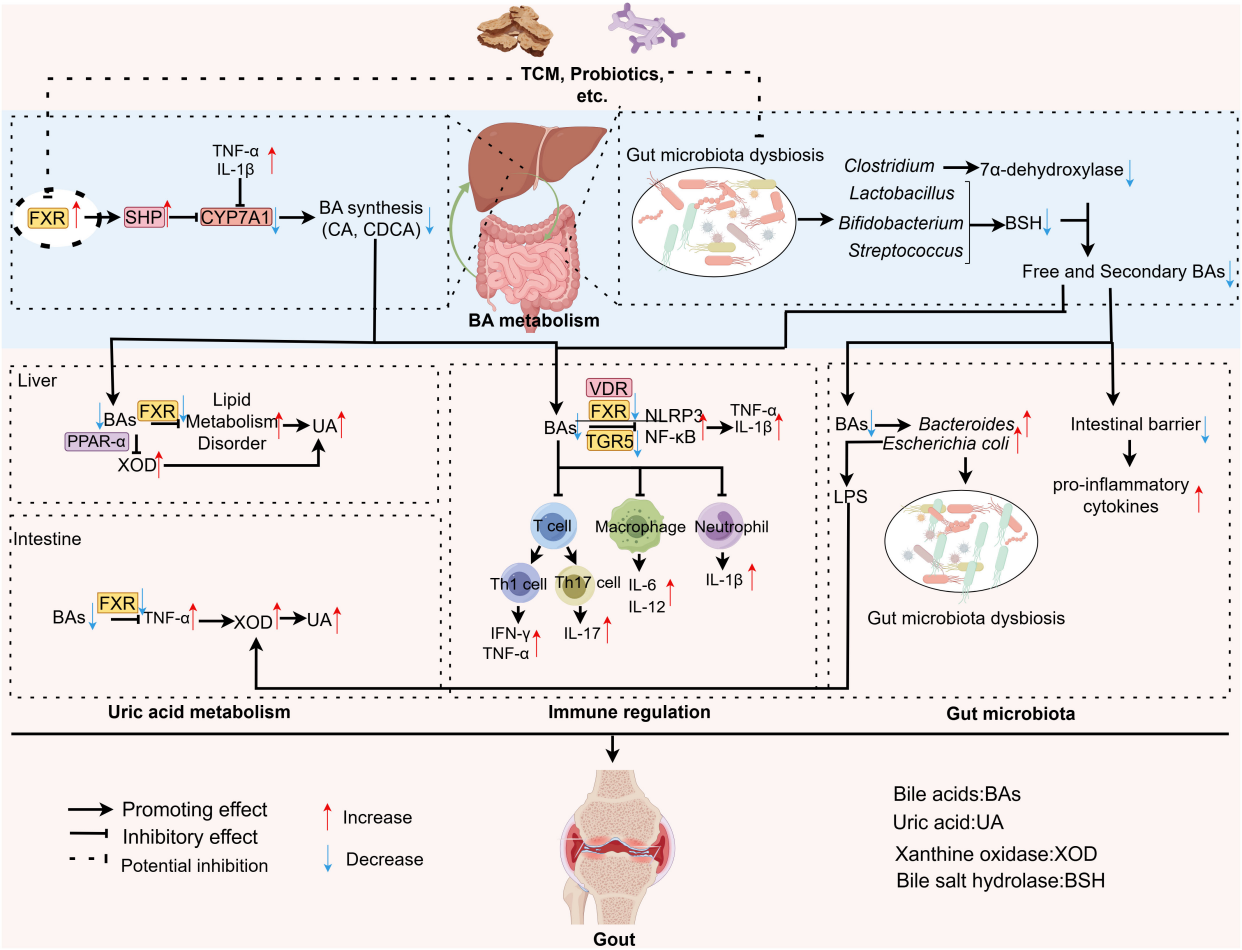
A mechanistic diagram summarizing the interactions of BA and UA metabolism, immune regulation, gut microbiota, and gout. This diagram outlines the causes of BA metabolic disorders in gout. Abnormal hepatic FXR activation lowers BA synthesis, while gut microbiota disruption reduces free and secondary BAs. Additionally, IL-1β and TNF-α further decrease bile acid production. Together, these factors reduce BA levels in gout, negatively impacting UA metabolism, immune regulation, and gut microbiota. UA Metabolism: Reduced BA levels can lead to increased expression or activity of xanthine oxidase in both the liver and intestine, as well as disrupt lipid metabolism, ultimately resulting in excessive uric acid productio; Immune Regulation: Decreased BA levels diminish the activity of FXR, TGR5, and VDR receptors, thereby weakening their inhibitory effects on NLRP3 and NF-κB. Concurrently, they modulate immune cells, such as altering T cell differentiation and promoting neutrophil infiltration, which collectively exacerbates the inflammatory response. Gut Microbiota: Reduced levels of free and secondary BAs promote the proliferation of gram-negative bacteria (e.g., *Escherichia coli)* and compromise intestinal barrier integrity. This, in turn, fosters increased UA production and enhances the inflammatory response. In summary, BA metabolic dysregulation exacerbates the progression of gout by collectively impacting UA metabolism, immune regulation, and the gut microbiota. FXR, Farnesoid-X-receptor; SHP, Small heterodimer partner; IL-1β, Interleukin-1β; TNF-α, Tumor Necrosis Factor-α; IL-6, Interleukin-6; UA, Uric acid; VDR, Vitamin D receptor; TGR5, Takeda G protein-coupled receptor 5; XOD, Xanthine oxidase; BAs, Bile acids. PPAR-α, Proliferator-activated receptor alpha; IFN-γ, Interferon-γ; IL-12, Interleukin-12; IL-17, Interleukin-17; CYP7A1, Cholesterol 7 alpha-hydroxylase; NLRP3, NOD-, LRR- and pyrin domain-containing protein 3; LPS, Lipopolysaccharide; CA, Cholic acid; CDCA, Chenodeoxycholic acid.

Research indicates that compared to conjugated BAs, free BAs exert stronger inhibitory effects on bacterial growth, with significantly greater potency against gram-negative bacteria than gram-positive bacteria (99). A reduction in free BAs in gout promote the proliferation of gram-negative pathogens, such as *Escherichia coli* and *Bacteroides*. LPS, a key component of Gram-negative bacterial cell walls, potentiates both the synthesis and activity of XOD, leading to the excessive production of UA and exacerbating gout progression (26, 100). Additionally, secondary BAs exhibit strong antimicrobial activity (93, 94), and exert protective effects on gout by suppressing inflammatory responses through TGR5-mediated downregulation of the NF-κB signaling pathway (101). In gout, the levels of secondary BAs decrease, compromising intestinal barrier integrity and increasing the risk of intestinal inflammation (102). This compromised barrier function facilitates the translocation of LPS into systemic circulation. Consequently, LPS activates the TLR4/NF-κB inflammatory signaling pathway, leading to the upregulation of pro-inflammatory cytokines (103) and precipitating gout attacks (**Figure 5**).

This chapter investigates the mechanisms underlying BA metabolic dysregulation in gout. The aberrant activation of hepatic FXR in gout is identified as a significant contributor to the reduction in BA synthesis. some studies suggest that pro-inflammatory cytokines IL-1β and TNF-α suppress the expression of CYP7A1 and NTCP, leading to decreased BA synthesis and a reduction in the hepatic-enterohepatic circulation rate, thereby contributing to BA metabolic disorders. Moreover, gut microbiota dysbiosis in gout results in a reduction of microbial populations responsible for producing BSH and 7α-dehydroxylase. This reduction leads to a low ratio of free to secondary BAs, thereby altering the BA pool. The combined effects of decreased BA synthesis and an altered BA pool decrease the activities of FXR, TGR5, and VDR, which subsequently induce or promote inflammatory responses, thereby exacerbating the progression of gout. Building upon the previously discussed factors contributing to BA metabolic disorders, we aim to identify and assess potential therapeutic agents for gout by modulating BA metabolism.

## 4 Potential therapeutic agents for gout treatment that target BA metabolism

### 4.1 FXR antagonists

FXR antagonists ameliorate reduced BA synthesis in gout by inhibiting aberrant activation of FXR. BA synthesis is regulated through two primary pathways: the FXR/SHP pathway and the FXR/FGF19/FGFR4 pathway. Blocking both FXR-mediated pathways with FXR antagonists upregulates the expression of CYP7A1, thereby increasing BA synthesis and fundamentally addressing the impaired synthesis observed in gout (104). Both UDCA and glycine-conjugated ursodeoxycholic acid (GUDCA) function as FXR antagonists (105). UDCA can be used in cases where BA production is impaired, and it has anti-inflammatory properties (14). It can inhibit the growth of the pathogenic bacterium *Clostridium difficile*, reduce the damage caused by the pathogen to intestinal epithelial cells, improve intestinal barrier damage, and suppress inflammatory responses (106) and the occurrence of gout. GUDCA selectively antagonizes intestinal FXR signaling, ameliorates insulin resistance (107), enhances renal UA excretion, and reduces serum urate levels - thereby exerting therapeutic effects against gout. FXR antagonists represent potential therapeutic agents for treating gout through the regulation of BA metabolism (as summarized in **Table 1**).

**Table 1:** Potential therapeutic agents targeting bile acid metabolism in gout: FXR antagonists and TGR5 agonists.

Drug type	Agent	Targeted diseases	Studied cohorts or animal models	Refs
FXR Antagonists	Ursodeoxycholic acid	Primary Biliary Cholangitis	Patients with Primary Biliary Cholangitis	(108)
	Ursodeoxycholic acid	​Cholestatic liver disease​	CD1 mice	(109)
	Glycoursodeoxycholic acid	Insulin Resistance	Intestine-specific *Fxr* knockout mice (*Fxr^ΔIE^*) and floxed *Fxr* control mice (*Fxr^fl/fl^*) under high-fat diet feeding	(107)
	Glycoursodeoxycholic acid	Type 2 Diabetes Mellitus	db/db mice	(110)
	Glycoursodeoxycholic acid	Rheumatoid arthritis	Collagen-Induced Arthritis Rats	(111)
	7-keto-lithocholic acid	Intestinal injury	Intestinal Organoids	(112)
	Mebhydrolin	Type 2 Diabetes Mellitus	HFD / STZ-induced type 2 diabetes mellitus mice	(113)
	Stigmasterol	Steatohepatitis	High-fat/high-cholesterol diet-fed mice	(114, 115)
	Guggulsterone	Hyperlipidemia	Wild-type and FXR-null mutant mice were fed a normal diet or a high-cholesterol diet	(116)
TGR5 Agonists	6α-ethyl-23(S)-methyl-cholic acid(6-EMCA, INT-777)	Atherosclerosis	*Ldlr ^- / -^ Tgr5 ^+ / +^* and *Ldlr ^- / -^ Tgr5 ^- / -^* mice	(117)
	6α-ethyl-23(S)-methylcholic acid (6-EMCA, INT-777)	Obesity and Diabetic Kidney Disease	Obesity mice / Diabetic mice	(118)
	3-oxolithocholic acid (3-oxoLCA)	Rheumatoid arthritis	CD4^+^ T cell / macrophages / CIA mice	(119)
	isolithocholic acid (isoLCA)	Rheumatoid arthritis	CD4^+^ T cell / macrophages / CIA mice	(119)
	isolithocholic acid (isoLCA)	Non-alcoholic Steatohepatitis	Mice with non-alcoholic steatohepatitis induced by a high-fat diet	(120)
	1,5-anhydroglucitol (1,5-AG)	Chronic Kidney Disease	Mice with renal fibrosis	(121)

### 4.2 TGR5 agonists

TGR5 agonists treat gout by promoting the excretion of UA and suppressing inflammation. The potent TGR5 agonist 6α-ethyl-23(S)-methylcholic acid (INT-777) inhibits inflammation in macrophages through TGR5-mediated targeting of cAMP and NF-κB signaling pathways (117). Furthermore, INT-777 induces the release of GLP-1 in enteroendocrine cells, ameliorating insulin resistance (122) and thereby promoting the excretion of UA. Additionally, the secondary bile acids 3-oxoLCA and isoLCA function as TGR5 agonists that suppress the differentiation of Th17 cells *in vitro* through two mechanisms: (1) directly inhibiting the expression of RORγt, and (2) promoting the polarization of M2 macrophages, which indirectly inhibits the development of Th17 cells (119). TGR5 agonists are promising therapeutic agents for treating gout (as summarized in **Table 1**).

### 4.3 Probiotics and prebiotics

Probiotics and prebiotics exert therapeutic effects on gout by promoting BA synthesis and reducing BA excretion. In a randomized controlled trial of 160 gout patients, the anti-inflammatory effects of probiotic supplementation were associated with BA-mediated inhibition of the activation of the NLRP3 inflammasome and reduced PGE2 expression via butaprost modulation (123). Wu et al. found that the administration of JS-3 in hyperuricemic quails increased the intestinal abundance of beneficial bacteria including *Bifidobacterium*, *Bacteroides unclassified_f-Lachnospiraceae*, and *norank_fynorank_o-Clostridia_UCG-014*, while reducing the abundance of pathogenic bacteria. This restored the diversity and function of gut microbiota and enhanced the production of α-cholic acid and ursodeoxycholic acid 3-sulfate, consequently alleviating hyperuricemia (124). Furthermore, prebiotics modulate BA metabolism, as evidenced by inulin increasing fecal concentrations of DCA and LCA in dogs (125). Insoluble dietary fiber from soybean hulls (a recognized prebiotic) significantly increases the abundance of *Bifidobacterium* and *Lactobacillus* abundance (126). Dietary fibers such as β-galactooligosaccharides reduce the fecal excretion of CA, α-rhamnolic acid, and DCA in mice (127). Thus, probiotics and prebiotics are promising therapeutic agents for correcting BA dysmetabolism in gout.

### 4.4 Traditional Chinese medicine

Traditional Chinese medicine (TCM) alleviates gout by modulating BA metabolism. *Astragalus membranaceus* restored the impairments structure of the microbiota in hyperuricemic mice by increasing the relative abundances of beneficial bacteria (Lactobacillaceae and *Lactobacillus murine*) but decreasing the relative abundances of pathogenic bacteria (Prevotellaceae, Rikenellaceae and Bacteroidaceae). This elevates levels of chenodeoxycholic acid, sulfolithocholylglycine, 3α,6β,7β-trihydroxy-5β-cholanic acid, and nutriacholic acid, thereby ameliorating metabolic disorders (128). *Phellinus igniarius*, a medicinal and edible fungus, modulates BA metabolism via its polysaccharides. It upregulates the expression of CYP8B1 and increases tauroursodeoxycholic acid (TUDCA) levels, which in turn inhibits the expression and activity of XOD, ultimately leading to a reduction in the production of UA (129). *Poria cocos* treats hyperuricemia by regulating BA metabolism, which involves an increase in chenodeoxycholic acid and a decrease in glycylcholic acid, restoring their balance (130). Atractylenolides II and III from *Atractylodes macrocephala* antagonize the FXR receptor, activating CYP7A1 to increase BA synthesis (131) and counter BA deficiency in gout. Therefore, TCM has broad therapeutic potential for gout management (**Table 2**).

**Table 2:** Potential therapeutic agents from traditional Chinese medicine for gout-induced bile acid metabolic dysregulation.

Drug name	Pharmacological mechanisms	Studied cohorts or animal models	Refs
*Astragalus membranaceus*	*(Lactobacillaceae*, *Lactobacillus murine*)↑ *and (Prevotellaceae, Rikenellaceae, Bacteroidaceae)↓ → (*chenodeoxycholic acid, sulfolithocholylglycine, 3α,6β,7β-trihydroxy-5β-cholanic acid, nutriacholic acid)↑	Hyperuricemic Mice	(128)
*Phellinus Igniarius*	TUDCA↑ *→* XOD*↓ →* UA↓	Hyperuricemic Mice	(129)
*Poria cocos*	CDCA↑; GCA↓	Hyperuricemia rat	(130)
*Atractylodes macrocephala*	FXR*↓ →* CYP7A1↑	–	(131)
Si-miao-yong-an decoction	Lipid accumulation*↓*; (CA, GCA, TCA)↑; classical neutral bile acid synthesis↑	Hyperlipidemia rat	(132)
Xiayuxue decoction	*(Bifidobacterium*, *Lactobacillus)*↑ and *(Oscillibacter)↓ →* primary bile acid levels↑	Subcutaneous Hepatocellular Carcinoma Xenografts in Nude Mice	(133)

↑, increase/upregulation; ↓, decrease/downregulation; →, “leads to” or “results in”; TUDCA, tauroursodeoxycholic acid; XOD, xanthine oxidase; UA, uric acid; CDCA, chenodeoxycholic acid; CA, cholic acid; GCA, glycocholic acid; TCA, taurocholic acid; CYP7A1, cholesterol 7alpha-hydroxylase.

This chapter primarily evaluates potential drugs for the treating of gout by targeting BA metabolism. Key approaches include, FXR antagonists, which mitigate the underlying issue of decreased BA synthesis by inhibiting FXR receptor activation; TGR5 agonists, which provide anti-inflammatory benefits through the activation of TGR5; prebiotics and probiotics, which address BA metabolic disorders by modifying the gut microbiota; TCM, which ameliorates BA metabolic disorders by restoring gut microbiota structure and enhancing BA synthesis. These approaches aim to treat gout by modulating BA metabolic disorders.

## 5 Challenges and prospects of clinical translation

The clinical application of TCM in the management of gout is extensive, highlighting its ability to alleviate symptoms and reduce side effects. Clinical research has demonstrated that the Huangqin Qingrechubi capsule significantly enhances lipid metabolism disorders and inflammatory responses in patients with gouty arthritis by modulating the lncRNA H19/APN/PI3K/AKT pathway, thereby effectively mitigating gout symptoms (134). Compared to conventional Western medicine, compound TCM formulations exhibit greater overall efficacy in the treatment of acute gouty arthritis. These findings indicate that TCM compounds significantly improve serum UA levels, erythrocyte sedimentation rates, and C-reactive protein levels while also decreasing the incidence of adverse reactions (135). These studies provide substantial evidence supporting the application of TCM gout in treatment. However, the multi-component nature of TCM makes the identification of bioactive components and the clarification of synergistic or antagonistic relationships among them particularly complex, which remains a significant challenge in TCM. Additionally, interest in the application of probiotics for gout management has increased in recent years. Probiotics fermented apple juice can ameliorate hyperuricemia by reversing gut microbiota dysbiosis and increasing the abundance of beneficial bacteria, such as *Lactobacillush*, *Faecalibaculum*, and *Lachnospiraceae_NK4A136_group (* 87). Moreover, a clinical trial revealed that, compared to the control group using febuxostat alone, the combination with Probio-X probiotics significantly reduced serum UA levels and the frequency of acute gout attacks. The study also highlighted that the efficacy of probiotics is highly individualized (123). Therefore, although probiotics hold broad prospects in gout treatment, further clinical research is still necessary to explore their potential in personalized therapy. Moreover, strategies aimed at targeting BA metabolism for disease treatment present promising application prospects. In a study focused on liver cancer therapy, the nanodelivery of BA receptor modulators significantly modified the immune microenvironment in mouse liver tumors, thereby inducing a robust anti-tumor immune response and offering a novel approach for the precise treatment of liver cancer (136). Similarly, the application of nanoemulsion-loaded obeticholic acid (OCA) to precisely manipulate liver sinusoidal endothelial cells enhances the secretion of chemokine ligand 16, which in turn activates natural killer T cell-mediated immunotherapy for liver cancer (137). Similarly, although the clinical translation of treating gout by regulating BA metabolism currently faces some challenges, such as the limited number of clinical studies on modulating BA metabolic disorders for gout treatment, as well as concerns regarding the safety and efficacy of this approach, which requires further clinical research, it provides a new method and approach for treating gout.

## 6 Conclusion and prospects

BAs are synthesized primarily in the liver through classical and alternative pathways (138), followed by microbial metabolism and transformation in the gut (58, 139). In the intestine, the gut microbiota converts primary BAs into secondary BAs (50, 62)(e.g., DCA, LCA, and UDCA) (52, 140). As pivotal metabolic regulators, BAs play an essential role in maintaining metabolic homeostasis. Gout, a clinically prevalent metabolic disorder, is characterized by inflammatory responses triggered by the deposition of MSU crystals in joints and surrounding tissues (141). Chronic recurrence of gout attacks can lead to joint deformities, renal impairment, and cardiovascular complications, posing a significant threat to human health (142). Dysregulation of BA metabolism occurs when any component of BA synthesis, secretion, or reabsorption, or microbial modification becomes impaired. Clinical studies have demonstrated pervasive BA metabolic disturbances in gout patients (29).

BA dysmetabolism is a critical pathological indicator of gout, actively promoting its onset and progression. Studies have demonstrated that BA synthesis is reduced and BA levels are decreased in gout. With respect to UA metabolism, BAs downregulate XOD mRNA expression via the inhibition of PPARα receptor, thereby reducing UA production. Consequently, decreased BA levels in gout increase serum urate concentrations (22). Furthermore, decreased BA availability impairs FXR activation, disrupting lipid metabolism and reducing UA excretion. Moreover, intestinal FXR deficiency increases intestinal XOD activity, resulting in higher uric acid production. ultimately inducing hyperuricemia. Concerning immune responses, weakened activation of FXR and TGR5 via the depletion of BAs increases the levels of pro-inflammatory cytokines, amplifying inflammatory cascades. BAs also suppress the differentiation of T-cells into Th17 cells (reducing inflammatory cytokine secretion), neutrophil infiltration, and macrophage-driven inflammation. Thus, reduced BA levels in gout exacerbate inflammatory responses, accelerating disease progression. Regarding gut microbiota, BAs actively shape its composition and structure, thereby modulating UA metabolism and inflammatory responses to and contributing to the progression of gout. To date, the pathogenesis of gout primarily involves multiple aspects including disorders of purine and UA metabolism, inflammatory responses, and gut microbiota dysbiosis, with BA metabolism permeating these processes and playing a crucial role in the onset and progression of gout.

This review further elucidates the mechanisms underlying BA dysmetabolism in gout. In the gout state, aberrant FXR activation and pro-inflammatory cytokines (IL-1β, and TNF-α) suppress BA synthesis. Some studies suggest that the pro-inflammatory cytokines IL-1β and TNF-α also suppress the expression of NTCP, and impaired function of NTCP affect BA transport. Moreover, the gut microbiota plays a pivotal role in BA metabolism. In gout, gut dysbiosis reduces microbial populations producing BSH and 7α-dehydroxylase, thereby decreasing the proportion of free and secondary BAs. The subsequent decline in the production of these free and secondary BAs leads to increased UA production and compromises intestinal barrier, ultimately contributing to gout pathogenesis. Consequently, the dysregulation of BA metabolism in gout may stem from disruptions in BA biosynthesis, transport systems, and the composition of the gut microbiota.

Based on the underlying causes of BA metabolism abnormalities in gout, in this review, we summarized several potential therapeutic drugs for treating BA metabolic disorders in gout. Although preclinical studies have elucidated the mechanisms by which drugs can treat gout through modulation of BA metabolism, their efficacy still requires validation in large-scale clinical trials. Given the complex interplay between BA metabolism and gut microbiota, treatments such as TCM and probiotics for gout may lead to variable therapeutic outcomes due to individual differences in gut microbiota. Future studies should focus on an in-depth investigation the gut microbiota-BA relationship to develop more precise probiotic formulations and TCM compounds, thereby enhancing treatment efficacy. Moreover, owing to the widespread distribution of FXR/TGR5 receptors in the human body, future targeted therapies for these receptors should explore advanced drug delivery technologies to develop more precise targeted formulations that minimize adverse effects. For example, in the treatment of hepatocellular carcinoma, researchers have designed a polyoxazole-based nanosystem for the delivery of OCA and 5β−CA to the liver to minimize the adverse effects of these BA modulators (136).

In conclusion, BA metabolism dysregulation contributes significantly to the pathogenesis of gout. The improvement of BA dysregulation holds promise as a potential therapeutic target or pathway for preventing and treating gout. However, considering the existing constraints in clinical gout management and the paucity of research regarding both the involvement of BA abnormalities in gout progression and the therapeutic efficacy of BA-targeted interventions, it is imperative that future studies should aim to elucidate the underlying molecular mechanisms of BA metabolism in gout. Additionally, conducting relevant clinical trials is essential. Such research endeavors may reveal novel pathways and strategies for the treatment of gout.
